# Resistance of Tick Gut Microbiome to Anti-Tick Vaccines, Pathogen Infection and Antimicrobial Peptides

**DOI:** 10.3390/pathogens9040309

**Published:** 2020-04-22

**Authors:** Agustín Estrada-Peña, Alejandro Cabezas-Cruz, Dasiel Obregón

**Affiliations:** 1 Faculty of Veterinary Medicine, University of Zaragoza, 50013 Zaragoza, Spain; 2 Group of Research on Emerging Zoonoses, Instituto Agroalimentario de Aragón (IA2), 50013 Zaragoza, Spain; 3 UMR BIPAR, INRAE, ANSES, École Nationale Vétérinaire d’Alfort, Université Paris-Est, 94700 Maisons-Alfort, France; 4 Center for Nuclear Energy in Agriculture, University of São Paulo, Piracicaba, São Paulo 13400-970, Brazil; 5 School of Environmental Sciences, University of Guelph, Guelph, ON N1G 2W1, Canada

**Keywords:** *Ixodes scapularis*, microbiota, disturbance, functional redundancy, core microbiome

## Abstract

*Ixodes scapularis* ticks harbor microbial communities including pathogenic and non-pathogenic microbes. Pathogen infection increases the expression of several tick gut proteins, which disturb the tick gut microbiota and impact bacterial biofilm formation. *Anaplasma phagocytophilum* induces ticks to express *I. scapularis* antifreeze glycoprotein (IAFGP), a protein with antimicrobial activity, while *Borrelia burgdorferi* induces the expression of PIXR. Here, we tested the resistance of *I. scapularis* microbiome to *A. phagocytophilum* infection, antimicrobial peptide IAFGP, and anti-tick immunity specific to PIXR. We demonstrate that *A. phagocytophilum* infection and IAFGP affect the taxonomic composition and taxa co-occurrence networks, but had limited impact on the functional traits of tick microbiome. In contrast, anti-tick immunity disturbed the taxonomic composition and the functional profile of tick microbiome, by increasing both the taxonomic and pathways diversity. Mechanistically, we show that anti-tick immunity increases the representation and importance of the polysaccharide biosynthesis pathways involved in biofilm formation, while these pathways are under-represented in the microbiome of ticks infected by *A. phagocytophilum* or exposed to IAFGP. These analyses revealed that tick microbiota is highly sensitive to anti-tick immunity, while it is less sensitive to pathogen infection and antimicrobial peptides. Results suggest that biofilm formation may be a defensive response of tick microbiome to anti-tick immunity.

## 1. Introduction

*Ixodes scapularis* is an important vector of *Borrelia burgdorferi* and *Anaplasma phagocytophilum*, causal agents of Lyme disease and human granulocytic anaplasmosis, respectively [1]. *Borrelia burgdorferi* is an extracellular bacterium and *A. phagocytophilum* is an obligate intracellular bacterium. However, both bacteria are maintained in a mammal-tick infectious cycle that involves intimate interactions with ticks during the colonization of its gut, and subsequent migration to the salivary gland, before transmission to the vertebrate host [1]. *Ixodes scapularis* harbors a diverse group of native microbes, ranging from viruses to bacteria [2]. These microbial communities influence the ability of *B. burgdorferi* and *A. phagocytophilum* to colonize and persist within the vector [3,4,5]. Microbial biofilms [6], generated by diverse bacterial species, are essential for bacterial colonization and successful symbiotic relationships in arthropod hosts [7,8]. *Borrelia burgdorferi* and *A. phagocytophilum* colonization in *I. scapularis* is associated with changes in the taxonomic composition of the microbiota resulting in diffuse biofilms [3,4]. Tick gene expression is affected by *B. burgdorferi* and *A. phagocytophilum* infection in *I. scapularis* gut. A secreted protein with a Reeler domain (PIXR) and an antifreeze glycoprotein (IAFGP) with antimicrobial properties are upregulated by *B. burgdorferi* and *A. phagocytophilum*, respectively [3,4,9]. The IAFGP and the anti-PIXR immunity also alter the tick microbiota and result in diffuse and dense biofilms, respectively [3,4].

**Figure 1:**
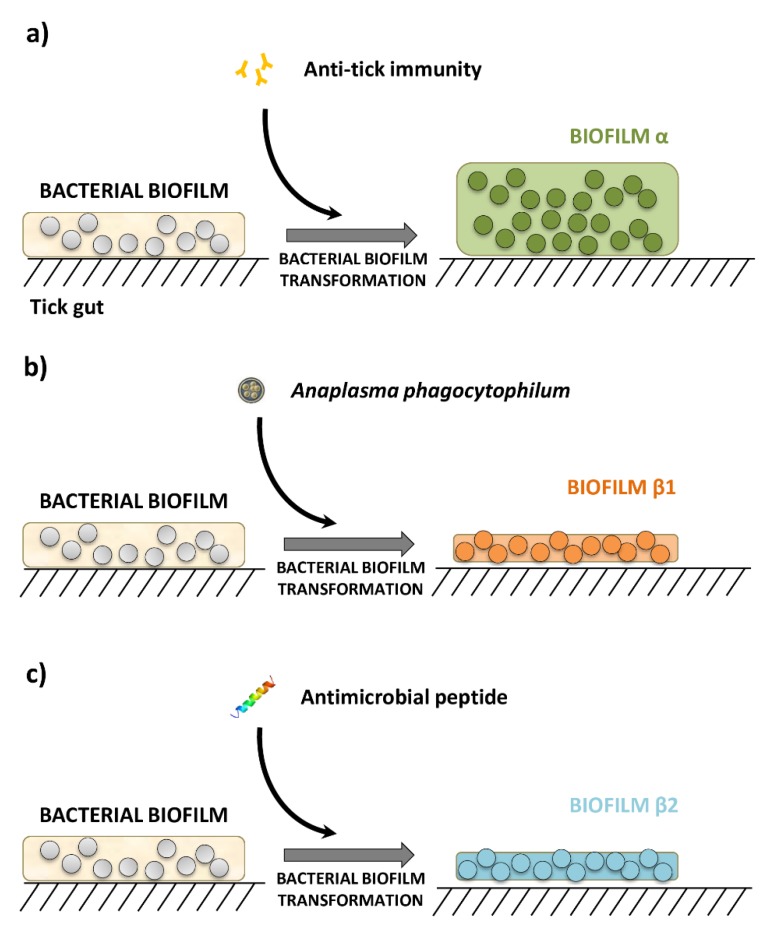
Schematic diagram of three different microbial communities resulting from different disturbance factors. (**a**) Anti-tick immunity results in a microbial community that increases biofilms in the tick gut [4], referred hereafter as biofilm α, (**b**) disturbance of tick gut microbial communities by *A. phagocytophilum* infection [3], and **c)** antimicrobial peptide treatment [3,9], produces scattered and fragmented bacterial biofilms, refereed hereafter as biofilms β1 and β1, respectively.

Based on these evidences, we asked to what degree exposure to pathogens, antimicrobial peptides or anti-tick immunity would impact the resistance of tick microbiome. We hypothesized that these disturbing factors induce taxonomic and functional changes that impact the resistance of tick microbiota, and may explain the properties of two set of biofilms that we named α and β (Figure 1). 

To test this hypothesis, published 16S rRNA gene amplicon sequence datasets obtained from tick microbiota upon disturbance by *A. phagocytophilum* [3], antimicrobial peptide IAFGP [3] and anti-tick immunity specific to PIXR [4] were used for the annotation of taxonomic profiles and prediction of functional traits of the microbiome, using the state-of-the-art metagenomics tool PICRUSt2 [10]. The taxonomic and functional profiles were quantified and compared in terms of abundance, diversity and composition, parameters used to measure the resistance of microbial communities to disturbance [11,12]. The taxonomic and functional structure of the microbial communities was then quantified and compared using co-occurrence networks. Resistance to taxa extinction and connectedness loss was tested on the taxonomic networks by the systematic removal of taxa in random and directed manners. A large effect on resistance was considered when all taxonomic and functional parameters of abundance, diversity, composition and resistance to taxa extinction were affected by disturbance. Here, we show that anti-tick immunity has a large impact on the tick microbiome, enhancing both taxonomic and functional diversity. Microbial community response to anti-tick immunity produced the over-representation of pathways involved in biofilm formation. In contrast, tick microbiota is less sensitive to *A. phagocytophilum* and antimicrobial peptide, since they alter the taxonomic composition, but not the pathway profile of the microbiome. We conclude that anti-tick immunity is a major disruptor of tick microbiota, favoring the abundance of strong biofilm formers. Results demonstrate that communities of bacteria are functionally redundant, suggesting a mechanism by ticks selecting the adequate microbiome fulfilling a core set of functions.

## 2. Methods

### 2.1. Original Data Sets

We used published 16S rRNA gene (16S) sequencing datasets. The original studies described the taxonomic composition of the gut microbiomes of larvae and nymphs of *I. scapularis* ticks subjected to disturbance by anti-tick immunity, pathogen infection or the treatment with an antimicrobial peptide (detailed below). These datasets were generated by the same research group using an *I. scapularis* tick colony and comparable methodologies. A total of 251-base paired-end reads were generated by amplicon sequencing of the V4 variable region of the bacterial 16S gene, using barcoded universal primers (515F/806R) and sequenced on an Illumina MiSeq system. The raw data were obtained in the frame of three different experiments:

i) The study by Narasimhan et al. [4] reported that the infection of larvae of *I. scapularis* with *B. burgdorferi* increased the expression of the tick protein PIXR, which facilitates *B. burgdorferi* infection and molting of larvae. The composition of the microbiota of tick larvae fed on C3H/HeJ mice immunized with recombinant PIXR and infected with *B. burgdorferi* was compared with that of ticks fed on C3H/HeJ mice immunized with Ovalbumin (OVA, an antigen not found in ticks) and infected with *B. burgdorferi*. The authors also showed that anti-tick immunity specific to PIXR induces the formation of dense bacterial biofilms in the tick gut. These two 16S datasets are hereinafter referred to as ‘Bb-anti-PIXR’ (n = 16) and ‘Bb-anti-Ova’ (n = 24). Comparisons were used to represent the microbiota composition in response to anti-tick immunity as disturbing factor;
ii) The report by Abraham et al. [3] studied the changes in gut microbiota composition and biofilms of nymphs of *I. scapularis* fed on *A. phagocytophilum*-infected or not infected C3H/HeJ mice. These two 16S datasets are hereinafter referred to as ‘Ap-infected’ (n = 20) and ‘Ap-uninfected’ (n = 10). Comparisons between them were used to assess the microbiota composition in response to the disturbing factor *A. phagocytophilum* infection;
iii) In addition, Abraham et al. [3] showed that *A. phagocytophilum* induces ticks to express an anti-freeze glycoprotein (IAFGP), an antimicrobial protein with the ability to alter microbiota composition [3] inducing the formation of scattered and diffused bacterial biofilms [9]. A derivative peptide of IAFGP, P1 (PARKARAATAATAATAATAATAAT) affect the tick gut microbial community compared with a scrambled P1 (sP1) peptide (AATAATATAAARRAAAAPTTAKTT) [3]. These two 16S datasets are hereinafter referred to as ‘P1’ (n = 16) and ‘sP1’ (n = 12) and represents an example of microbiota composition in response to antimicrobial peptides.

### 2.2. Processing of Original Raw Sequences

We performed de novo taxonomic annotation of all the 16S datasets. To this end, the sequences were downloaded from SRA repository [13], extracted, and de-interlaced in two fastq datasets containing only the first or second mate read [14] using the data analysis platform Galaxy (http://usegalaxy.org). Demultiplexed fastq files were pre-processed and analyzed using QIIME2 pipeline (v. 2019.1) [15]. Briefly, the DADA2 software package [16] was used (via q2-dada2) for denoising the fastq files and merging mate reads. All amplicon sequence variants (ASVs) were aligned with MAFFT [17] (via q2-alignment) and used to construct a phylogeny with FastTree 2 [18] (via q2-phylogeny). Alpha diversity metrics (i.e., Faith’s phylogenetic diversity index [19] and Pielou’s evenness index [20]) and beta diversity metrics (i.e., weighted- and unweighted UniFrac [21]) were estimated using the q2-diversity plugin, after samples were rarefied (subsampled without replacement). Taxonomy was assigned to ASVs using the q2-feature-classifier [22] classify-sklearn naìve Bayes taxonomy classifier against the 16S SILVA database (release 132) [23], trained 99%, hence only the target sequences fragment was used [24]. QIIME2 taxa barplot functions were used for viewing the taxonomic profiling by samples. The analyses of alpha and beta diversity were performed by Kruskal-Wallis and PERMANOVA statistical tests, respectively. For taxa comparisons, abundances based on all obtained reads were used, compared between datasets in each experiment by linear discriminant analysis effect size (LEfSe) method [25].

### 2.3. Prediction of Functional Traits in Tick Microbiome

The 16S amplicon sequences from each experiment were used to predict the metabolic profiling of each sample. PICRUSt2 [10], a robust bioinformatic tool, was used to predict the genomes of 16S amplicon sequences. The AVSs were placed into a reference tree (NSTI cut-off value of 2) that contained 20,000 full 16S sequences from prokaryotic genomes, which was then used to predict individual gene family copy numbers for each AVS. The predictions are based on several gene family catalogs: the Kyoto Encyclopedia of Genes and Genomes (KEGG) orthologs (KO) [26], Enzyme Classification numbers (EC) and Clusters of Orthologous Genes (COGs) [27]. Pathway abundances was inferred, based on structured pathway mapping based on MetaCyc database [28].

The resulting functional profiles were explored by alpha and beta diversity metrics (via q2-diversity) and compared between datasets by Kruskal-Wallis and PERMANOVA tests, respectively. To identify pathways with differential abundance between groups (control vs disturbed), functional profiles were compared by Welch’s t-test with multiple test correction by the Benjamini–Hochberg FDR (false discovery) method. The analysis was performed with the Statistical Analysis of Metagenomic Profiles (STAMP) software package [29], and visualized as a volcano plot. Samples from all the groups were compared by the Gneiss differential abundance test [30].

### 2.4. Taxonomic and Functional Co-Occurrence Networks and Network Resistance Analysis

The taxonomic profiling resulting from each dataset was used to build co-occurrence networks of interacting organisms [31]. We used the SparCC method [31] implemented in the R programming environment to produce the correlations of the abundance among bacterial genera. In taxonomic networks, the strength of correlation between pairs of bacterial genera is the weight of the link between any two co-occurring bacterial genera. Similar methods were used to build functional networks of the pathways profile of the microbiomes for each dataset, as assigned by PICRUSt2. The functional networks were built using pathways co-occurrence, with weight of the links derived from the correlations between any two pathways, according to feature abundance profile. Taxonomic and functional networks were built for each experimental condition ‘Bb-anti-Ova’, ‘Bb-anti-PIXR’, ‘Ap-uninfected’, ‘Ap-infected’, ‘sP1’ and ‘P1’.

Several indexes give an indication of topology and strength of the networks. These included the number of nodes and edges, weighted degree (WD), diameter of the network (a measure of its cohesiveness), modularity (an index that computes how interacting nodes arrange in modules), and the clustering coefficient (a measure of the density of each network). Changes in these indexes can explain the robustness of the network, the density of its links, or the nodes that tend to co-occur more frequently. Modularity maximization was used to extract modules from the networks [32]. Most of the calculations were done with the software Gephi 0.9.2 [33]. 

Attack tolerance was tested only on the taxonomic networks. The purpose was to measure their resistance to systematic removal of nodes, either by a random attack with 100 iterations, or by a directed attack, removing the nodes according to its value of centrality (the highest, the first). The number of secondary removals (i.e., nodes that became disconnected from the network after each node removal) was also calculated. The analyses of the networks resistance were done with the package NetSwan [34] for R.

To test the functional redundancy of tick microbial communities, we selected the top three bacterial modules within each taxonomic network. These modules included not less than 75% of the total bacterial genera detected in the network. The ASVs corresponding to each module were filtered in the raw dataset using q2-taxa plugin (13). The functional predictions for each module were produced and the functional profiles compared among modules using non-metric multidimensional scaling (NMDS), based on the Bray-Curtis distance. 

### 2.5. Identification of the Core Microbiome

We conducted a step-forward analysis to detect and characterize the taxonomic or functional cores of the *I. scapularis* microbiome. They were defined as the set of bacterial genera or metabolic pathways that were shared by most of the individual ticks in all datasets. First, we used a Venn diagram to identify the features present in at least one sample in each dataset. The second method was based on identifying the core features which persisted across serial fractions of the samples, performed with the Qiime2 plugin feature-table (core-features) in [15]. Finally, we integrated presence with feature abundance and connectedness to better characterize the core microbiome. We used a set of fuzzy logic rules [35] to calculate the probability of each bacterial genera or pathway to belong to the taxonomic or functional core. Fuzzy logic rules are not binary, but a range of probabilities. The maximum probability 1 indicates that a genus or a pathway ‘completely belongs’ to the core, while decreasing probabilities indicate a smaller probability to belong to the core. Fuzzy logic rules were built using the software Manifold v.8 (http://www.manifold.net). The degree of membership of a member to the core (either taxonomic or functional) was defined by the rules: (i) appears in the highest percent of ticks of the dataset, (ii) the number of reads is over the median of the distribution of reads detected in the dataset, (iii) the WD and centrality of the member is over the median of all the co-occurring members of the dataset, and (iv) the conditions before are not affected under disturbance. A member will have a maximum probability to be part of the core when all the conditions above are met simultaneously. As one or several of the rules above are not met, the probability of membership decreases. 

## 3. Results

### 3.1. Effect of Biological Disturbance on Taxonomic and Functional Profiles of Tick Microbiome 

**Figure 2:**
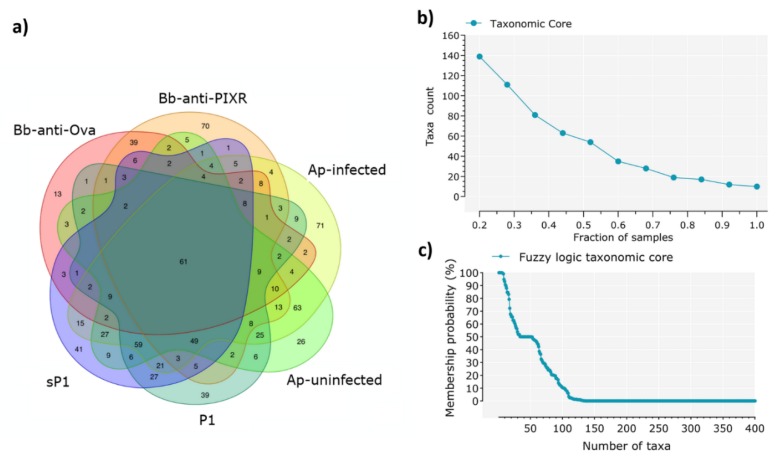
Identification of the taxonomic core. (**a**) Venn diagrams showing the sharing of bacterial taxa among the six experimental groups of gut microbiotas from larvae and nymphs of *I. scapularis* under disturbance factors, each data set includes microbial genera that were found in at least one sample of the experimental groups. (**b**) Identification of the taxonomic core across all the samples under study, a total of 928 taxa (genera) were found, among them, only 10 genera were shared for all the samples (98 samples). (**c**) The core taxonomic microbiome according to the fuzzy logic rules stated in Material and Methods. The number of taxa that belong to the core microbiome of the 98 samples decreases quickly: at the 96% of probability membership, only 50% of taxa remain.

Tick microbiota showed different taxonomic composition in the six 16S datasets (Figure S1a). Major differences were found between the tick stages (i.e., nymph and larvae). A Venn diagram analysis shows that each experimental group was associated to a set of specific bacterial genera that was not shared by the others (Figure 2a). 

A small taxonomic core of 61 bacterial genera (total: 821, 7.4%) was shared by at least one individual tick of each of the experimental groups (Figure 2a). The presence of a highly reduced taxonomic core was confirmed by taxa counting after progressive sampling, since 20% and 100% of all samples (n = 98) shared only 140 (total: 821, 17.1%) and 10 taxa (1.2%), respectively (Figure 2b). The only 10 taxa present in 100% of the samples were included in Table S1. The existence of a reduced taxonomic core was confirmed by fuzzy logic analysis, which showed that less than 50 bacterial taxa have a probability of membership higher than 50% (Figure 2c).

Community diversity indexes showed that anti-tick immunity produced a significant increase in phylogenetic diversity (Kruskal-Wallis test, *p* < 0.001) and species evenness (Kruskal-Wallis test, *p* < 0.001) (Figure S2a). However, *A. phagocytophilum* infection or the antimicrobial peptide did not modify the alpha diversity indexes (Figure S2b,c). Disturbing factors also changed the abundance of several taxa (Figure S3). Pairwise comparisons between datasets within the same experiment showed that anti-tick immunity, *A. phagocytophilum* infection and the antimicrobial peptide changed dramatically the abundance of specific bacterial genera (Figure S4). We observed that the abundance of 63 (total 78, 80.8%), 18 (total 46, 39.0%) and 22 (total 51, 43.1%) bacteria genera increased in response to anti-tick immunity, *A. phagocytophilum* infection and antimicrobial peptide, respectively. We asked whether biofilm α (Figure 1) was associated with an increase in the abundance of biofilm formers, while biofilms β1 and β2 (Figure 1) were associated with a reduction in the number of biofilm formers. The bacterial genera with higher abundance in response to anti-tick immunity include the strong biofilm formers *Mycobacterium* [36], *Tepidimonas* [37], *Rothia* [38] and *Leuconostoc* [39], whereas *A. phagocytophilum* infection and antimicrobial peptide reduced the presence of the biofilm formers *Gracilibacteria* [40] and *Enterococcus* [3], respectively.

**Figure 3:**
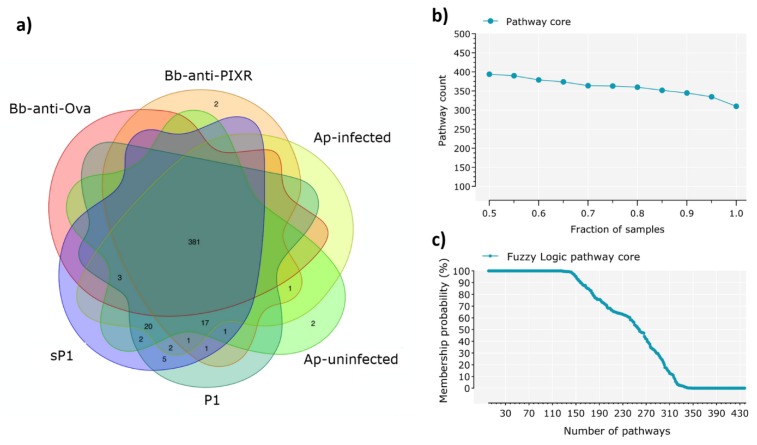
Characterization of the core metabolic pathway. (**a**) Venn diagrams showing the sharing of metabolic pathways among the six sample groups. Included are the pathways found in at least one sample of the group. (**b**) Identification of the pathway core across all the samples under study, a total of 440 pathways were found, of which 70% was found in all the samples (98 samples). (**c**) The core functional microbiome according to the fuzzy logic rules stated in Materials and Methods. The number of functions that belong to the core microbiome of the 98 samples remain stable, even at membership values as low as 68%, with 100% of functions belonging to the core functional microbiome at such low probability.

Based on the changes observed in the taxonomic composition and taxa abundance after disturbance, we expected a similar trend in the functional traits encoded in the tick microbiomes. Surprisingly, the Venn diagram analysis showed that all experimental groups shared the majority (i.e., 381) of the metabolic pathways (total: 437, 87.2%) and almost none of the disturbing factors was associated to a set of specific metabolic pathways (Figure 3a). 

Progressive sampling of the metabolic pathways (Figure 3b) and fuzzy logic analysis (Figure 3c) showed the persistence of a broad functional core despite disturbance. Metabolic pathways included in the functional core across all the samples are available in Table S2. 

We next asked whether this functional redundancy would be also observed at the bacterial community level. To evaluate this idea, we built networks of co-occurring bacteria (described in detail in the next section) and identified network modules representing bacterial communities. Network modules are formed by bacteria that co-occur more often with each other than with the other bacteria in the network and they can be considered as bacterial communities [41]. The three largest modules, including >75% of the network nodes, were selected in each network. Subsequently, we predicted and compared their pathway profiles. The results showed that the network modules in each network were similar in pathway content and abundance (Figure S5). These findings suggest that bacterial communities of tick microbiota are functional units containing a highly redundant set of metabolic pathways.

### 3.2. Disturbance Reshapes Co-Ocurrence Networks and Reduces Networks Tolerance to Taxa Extinction 

**Figure 4:**
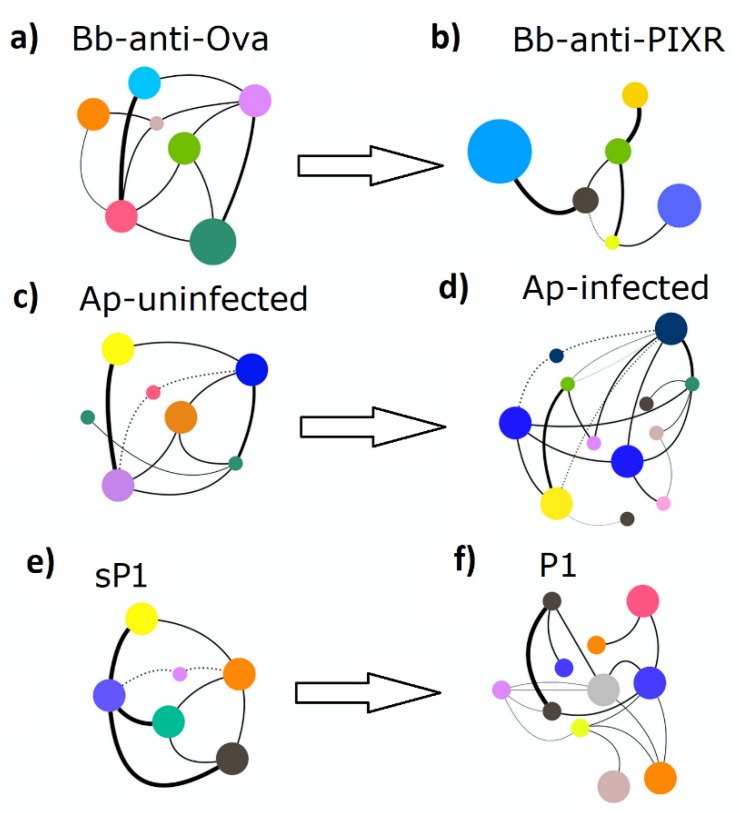
A schematic representation of the topological patterns of co-occurring microbial taxa in undisturbed and disturbed tick microbiota. The network of each experimental group: (**a**) Bb-anti-Ova, (**b**) Bb-anti-PIXR, (**c**) Ap-uninfected, (**d**) Ap-infected, (**e**) sP1, (**f**) P1 is presented. Circles (nodes) are bacterial genera and edges the co-occurrence between taxa. Colors are random, but circles with the same color mean for clusters of taxa that co-occur more frequently among them than with other taxa. The size of the circles and the labels proportional to the centrality (betweenness centrality) of each taxon in the resulting network (full networks provided in Figures S6–S8).

Bacteria co-occurrence networks were used also to quantify changes in topology and resistance to taxa extinction. Visual inspection of the taxonomic networks revealed that each disturbing factor changed the network topology (Figure 4), a result supported by their numerical features (Table 1). 

The topological changes observed in the co-occurrence networks of tick microbiota after disturbance indicate that anti-tick immunity, pathogen infection and antimicrobial peptide reshape the co-occurrence of microbial communities in the tick gut. Disturbance affects the connections between modules, explained by the average clustering coefficient. Anti-tick immunity resulted in a network with higher clustering coefficient, while *A. phagocytophilum* and the antimicrobial peptide reduced dramatically this property. Anti-tick immunity produced a network with two large modules and a higher number of nodes compared with the control (Figure S6). *Anaplasma phagocytophilum* completely altered the network by increasing the number of modules and decreasing the connection among them (Figure S7), properties that were similar in the antimicrobial peptide network (Figure S8). All the networks resulting from disturbed microbiota had higher diameters (increased laxity) than the controls.

To test the effect of disturbing factors on network resistance, the networks of co-occurring taxa were subjected to two different attack strategies: (i) random removal of nodes and (ii) directed removal of nodes starting from those with the highest centrality. In each case, we assessed the loss of connections and secondary extinctions in the network. Bacterial networks resulting from ticks exposed to anti-tick immunity (Figure S9a), *A. phagocytophilum* (Figure S9b) and anti-microbial peptide (Figure S9c) were less tolerant to both strategies of taxa removal. Every disturbance factor had an effect on the connectivity of the networks, reaching a 50% of disconnected taxa when 12%, 20% or 22% of taxa were removed for anti-tick immunity, *A. phagocytophilum* infection and anti-microbial peptide, against 30%, 24% and 23% removal in control groups, respectively. The anti-tick immunity induced a larger disconnection of the network than other disturbances to tick microbiota.

### 3.3. Anti-Tick Immunity Increases the Representation of Biofilm Formation Pathways in Tick Microbiome

We then measured the impact of disturbance on the metabolic traits of tick microbiome. We first compared the alpha and beta diversities of the metabolic pathways found in the microbiomes. Anti-tick immunity induced an increase in the pathway richness (Kruskal-Wallis test, *p* < 0.001) and evenness (Kruskal-Wallis test, *p* < 0.001), whereas *A. phagocytophilum* and the antimicrobial peptide had no significant effect on these alpha-diversity metrics (Figure S10). In agreement with this, the principal coordinate analysis (PCoA) of beta diversity showed that only anti-tick immunity produced a significant differentiation (PERMANOVA test, *p* < 0.001) in the functional profiles of the tick microbiome (Figure S10). *Anaplasma phagocytophilum* and the antimicrobial peptide produced minor and no change, respectively, on the functional diversity of tick microbiome (Figure S10).

**Figure 5:**
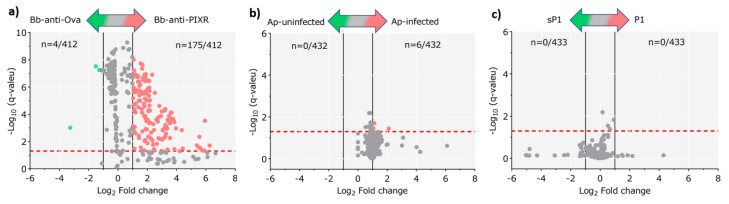
Differential functional profile of tick gut microbiome under different disturbance factors. Volcano plot showing differential pathway abundance between sample groups from the experiments: (**a**) anti-tick immunity, (**b**) *A. phagocytophilum* infection, and (**c**) antimicrobial peptide. The green and red dots indicate pathway (n) that display both large magnitude fold-changes and high statistical significance favoring disturbed and untreated groups, respectively, while the gray ones are not considered significant. The dashed red line represents the adjusted (Benjamini–Hochberg FDR method) *p*-value cutoff value of 0.05 (points above the line having *p* < 0.05 and points below the line having *p* > 0.05). The vertical black lines represent the log2 fold change absolute value cutoff of 1 (2-fold-change: log2 = 1).

Significant differences in the relative abundance of the metabolic pathways in response to the disturbing factors were observed only in ticks exposed to anti-tick immunity (Figure 5a). 

Despite the changes in the abundance of bacterial genera in response to *A. phagocytophilum* and antimicrobial peptide, these disturbing factors produced minor (Figure 5b) and no change (Figure 5c), respectively, on the abundance of metabolic pathways of the tick microbiome. A detailed comparison of metabolic pathways abundance across all the samples using the Gneiss test resulted in a dendrogram heatmap, showing that five clusters of functional categories explained the major differences among groups (Figure S11). The metabolic pathway composition of each cluster is available in Table S3. To evaluate the relative importance of each of these pathways, we built networks using pathway co-occurrence and used the weighted degree (WD) as a proxy of pathway importance. The functional network of tick microbiome exposed to anti-tick immunity showed higher connectivity than those of *A. phagocytophilum*-infected and antimicrobial peptide-treated ticks (Table 2). 

Disturbance of the tick microbiome changed the WD of metabolic pathways in the functional networks. Among the pathways with at least 2-fold change (log2 = 1) in WD, 87 (total 105, 82.9%) were over-represented in response to anti-tick immunity, while among the pathways with significant changes in response to *A. phagocytophilum* and antimicrobial peptide, 79 (total 87, 90.8%) and 74 (total 93, 79.6%) were under-represented, respectively.

**Figure 6:**
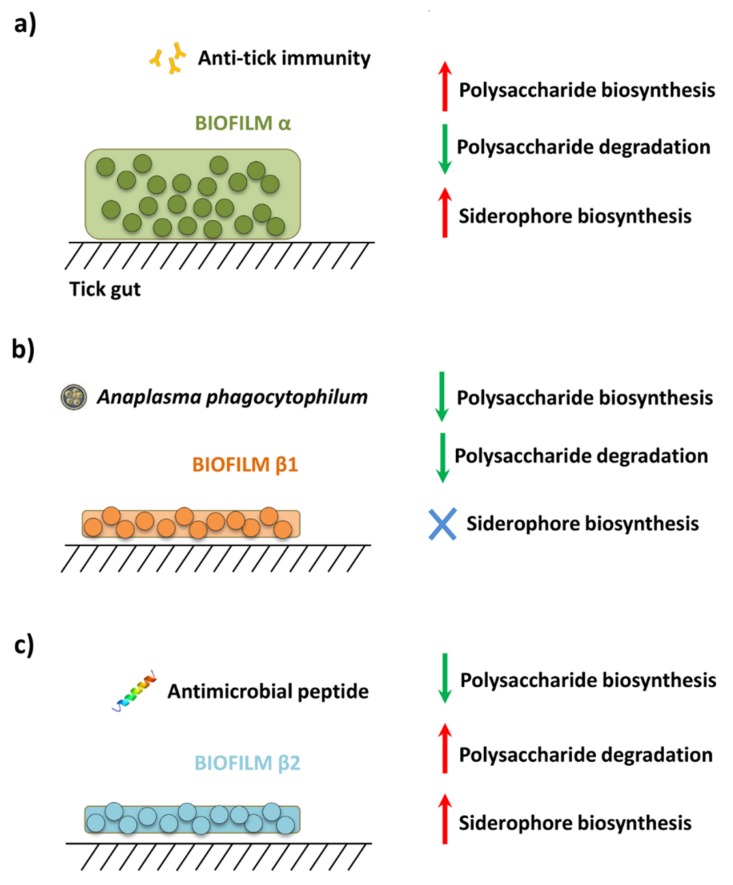
Biofilm pathways affected in response to disturbance. The biofilm formation pathways for which the weighted degree (WD) changed (2-fold change: log2 = 2) in response to the disturbing factors (**a**) anti-tick immunity, (**b**) *A. phagocytophilum* infection and (**c**) antimicrobial peptide are shown. WD values for all pathways are available in Figure S11. The increase and decrease in WD are shown by red and green arrow, respectively. Pathways for which the fold change was >2/<2 are represented by a blue cross.

The pathways with the greatest differences between groups (log2 ≥/≤ 2) are shown in Figure S12. Among the pathways with WD log2 ≥/≤ 2 in response to anti-tick immunity, three were related with polysaccharide biosynthesis, an essential process in biofilm formation. These pathways were colanic acid biosynthesis [42], peptidoglycan biosynthesis [43], and O-antigen biosynthesis [44]. With few exceptions, all the molecular components of these polysaccharide biosynthesis pathways were identified (Figure S13a). The relative abundance of these pathways was also found to increase significantly in response to anti-tick immunity (Figure S13b). Other pathways also involved in biofilm formation, including polysaccharide degradation [42] and siderophore biosynthesis [45,46], were also identified. Based on the comparison of WD values for the three group of pathways (i.e., polysaccharide biosynthesis, polysaccharide degradation and siderophore biosynthesis), a model of the functional profiles of biofilms α, β1 and β2 was proposed (Figure 6). 

## 4. Discussion

In this study, we tested the hypothesis that *I. scapularis* microbiome has different degrees of resistance to three disturbing factors, namely anti-tick immunity, pathogen infection and an antimicrobial peptide. To this end, we used 16S gene sequences available from previous publications [3,4]. These studies were originally aimed to elucidate the effects of anti-tick immunity specific to PIXR, *A. phagocytophilum* infection and antimicrobial peptide P1, on tick gut microbiota composition and bacterial biofilm formation [3,4]. Using recent advances on bioinformatics tools and benchmarks (i.e., Dada2 [16], and the classify-sklearn naìve Bayes taxonomy classifier [22]), our taxonomic analysis pipeline improved the accuracy in taxonomic classification of the sequences. This methodology allowed us to resolve each amplicon sequence variants (ASVs), instead of operational taxonomic units (OTUs). In addition, we incorporated the analysis of predicted metabolic profiling of the microbiomes, based on the novel bioinformatics tool PICRUSt2 [10]. Resistance could be defined as the degree to which the community withstands change in the face of disturbance [11]. To assess resistance of tick microbiota, we measured to what extent the taxonomic or functional profiles of the tick microbiome remained unchanged under different disturbing factors [12]. Furthermore, we explored the structure of microbiota by applying network analysis, a well-established methodology for detecting interactions within the microbiota [47,48]. A further test of resistance was performed on the taxonomic co-occurrence networks by quantifying the loss in connectivity and secondary extinctions due to taxa removal. 

The complex community of microbes living in the tick guts [2] plays an important role in pathogen colonization [3,4] and potentially on tick fitness. However, despite its presumed importance, the distinction between ‘constitutive’ and ‘transient’ tick microbiota has remained an elusive task. In this regard, a central question is whether a core constitutive microbiota consisting of bacterial groups common to all ticks exists [49]. The existence of a core microbiota has been previously addressed in terms of taxonomic composition [50,51]. Here we showed that the taxonomic core of *I. scapularis* is highly reduced, whereas the functional core is an important element of tick microbiome. We found evidence of a functional core in *I. scapularis* defined as 300 pathways present in 100% of the samples analyzed. However, the *I. scapularis* core microbiome was not defined by taxonomic associations, as only 10 taxa were shared by 100% of the samples. This suggests that microbial communities in ticks shared a set of metabolic pathways, regardless of the taxonomic identity of the microbes in tick microbiota. The existence of a functional core, confirmed at the level of bacterial communities, and the absence of a taxonomic core, points to functional redundancy (when multiple taxa contribute with the same metabolic function) as an emerging property of the tick microbiome.

Selected network modules can be considered as communities of bacteria with the highest frequency of co-occurrence [32]. The presence of different microbial communities in ticks suggest the existence of microbe–microbe interactions shaping the composition of tick microbiota. Little is known, however, about the driving forces behind these interactions and their effects on the dynamics of microbial communities. One hypothesis suggests that genetic and ecological factors influence tick symbionts [52] and/or strongly interconnected taxa to recruit other microbes to form a holobiont, the assemblage of different species (e.g., host and bacteria) that form an ecological unit [53]. Our results showed that the different network modules had similar metabolic traits which suggest that different bacterial communities within ticks share a functional core. A possible implication of such a property of the tick microbiome is to keep a functional stability independently of taxonomic changes caused by disturbance. This is consistent with the idea that the tick microbiome evolved through the recruitment of microbial populations of different taxa, but sharing a set of functions with possible impact on tick fitness [54,55].

*Ixodes scapularis* microbiota was modified by disturbing factors including anti-tick immunity, pathogen infection and antimicrobial peptide. Different mechanisms account for tick microbiota modulation by pathogens [3,4]. The rewiring of taxonomic networks after disturbance revealed the sensibility of tick microbiota to disturbance. Moreover, the taxonomic networks resulting after disturbance were less resistant to taxa removal. However, the only factor that had a large effect on bacterial diversity was anti-tick immunity. In accordance, the functional microbiome was affected only by anti-tick immunity, while the *A. phagocytophilum* infection and microbial peptide did not altered the pathway diversity of tick microbiomes. 

Immunity to PIXR induced a significant increase in microbial diversity and the abundance of biofilm formers which was associated with dense biofilms α (Figure 1a, [4]). However, the taxa removal test applied on the networks revealed that the microbial communities of ticks fed on animals immunized with PIXR were less resistant to taxa removal, suggesting that the microbial communities exposed to anti-tick immunity are less organized and less cohesive. This finding matched with the assumption that the proportion of interactions within a biological system modulates its stability, but it may become suddenly unstable as the system becomes larger [56,57]. This also suggests that anti-tick immunity could break the ecological balance within the tick gut and make tick microbial communities more susceptible to further stress. If the increase in microbial diversity induced by anti-tick immunity have a deleterious effect on the ecology of tick microbial communities, it could be then deduced that microbial communities can be tick specific and can accommodate only certain bacterial taxa. This is in agreement with a recent study showing a phylogenetic association between ticks and their microbiota across *Ixodes* ticks [55]. 

Anti-tick immunity also increased the functional diversity and the importance of pathways involved in biofilm formation including colanic acid biosynthesis, a major component of bacterial biofilms [58]. Biofilm creates a favorable environment that increases antibiotic resistance, impairs host immunity, and increases tolerance to nutritional deprivation [59,60,61]. The formation of biofilms α (Figure 1a) due to increase in biofilm formers may be part of a protective response of the tick microbiota to anti-tick immunity. This raises an interesting question: in addition to altering the ecology of tick microbial communities, can anti-tick vaccination trigger the formation of biofilms α that increase the resistance of ticks to vaccines? It is notable that PIXR is a negative regulator of biofilm’s formation [4]. Therefore, future studies should test whether immunity against tick proteins not related with biofilm formation would also induce the formation of biofilms α.

*Anaplasma phagocytophilum* and antimicrobial peptide were associated with diffuse biofilms (Figure 1b,c, [3]) and produced taxa replacement without impact on the microbial diversity metrics. However, *A. phagocytophilum* and antimicrobial peptide communities resulted in networks with lower susceptibility to taxa removal. The infection with *A. phagocytophilum* and the treatment with the antimicrobial peptide did not cause significant modifications on the diversity and abundance of the functional profiles of the tick microbiome, whereas the relative importance (measured as changes in WD) of several pathways changed in response to these disturbing factors. A common property of the microbiome disturbed by *A. phagocytophilum* and the antimicrobial peptide was a decrease in the importance of polysaccharide biosynthesis pathways (Figure 6b,c) which are important in the formation of bacterial biofilms [58,62,63]. Our results are consistent with previous reports of a decrease in the exopolysaccharide PNAG in tick gut infected with *A. phagocytophilum* [3]. The decrease of biofilms upon *A. phagocytophilum* colonization was suggested to be caused by the binding of IAFGP to D-alanine which blocks the formation of gram positive biofilms [3]. Our study suggests that the under-representation of biofilms forming pathways may be an additional mechanism by which *A. phagocytophilum* decreases tick guts biofilms.

## 5. Conclusions

This study demonstrated that anti-tick immunity has a large impact on the tick microbiome, enhancing both taxonomic and functional diversity. Results demonstrate that communities of bacteria in ticks are functionally redundant, suggesting a mechanism by which ticks select a microbiome fulfilling a core, and a specialized set of functions. We conclude that the functional core is an important component of the resistance of tick microbiome to disturbance. The functional core is redundant and is not defined or restricted by taxonomic composition. This is confirmed by the existence of a rich and redundant functional core, in opposition to a highly reduced taxonomic core. We strongly suggest to turn the classic taxonomic approach of comparing the tick microbiome between species into a functional framework. Despite functional redundancy in terms of composition, there were changes in abundance and importance of pathways that may have influenced the functional properties of the core microbiome in response to disturbance. Therefore, the functional core associated with the tick microbiome should be considered a dynamic entity. Biofilm formation pathways are part of the response of tick microbiota to disturbance. Tick microbiota is highly susceptible to anti-tick immunity, and the increase of biofilm formation pathways may be a response of tick microbiota to this disturbing factor. In contrast, tick microbiota is less sensitive to *A. phagocytophilum* colonization and tick antimicrobial peptide, which may reflect the coevolution and adaptation between tick-borne pathogens, the microbiota and the vector.

## Figures and Tables

**Table 1:** Topological features of the taxonomic networks under different disturbance factors.

Topological Features	Anti-Tick Immunity		*A. phagocytophilum* Infection		Antimicrobial Peptide	
	Bb-anti-OVA	Bb-anti-PIXR	Ap-uninfected	Ap-infected	sP1	P1
Nodes ^a^	204	329	416	506	378	364
Edges ^b^	2522	6974	11,170	43,389	7976	12,787
Modules ^c^	8	8	6	10	8	12
Network diameter ^d^	3	5	4	9	4	5
Average degree ^e^	24.73	26.82	53.71	17.15	42.20	26.29
Weighted degree ^f^	4.38	10.25	23.52	6.51	18.04	10.58
Clustering coefficient ^g^	0.393 (8313)	0.581 (34,613)	0.528 (113,571)	0.376 (12,102)	0.521 (61,799)	0.490 (22,053)

^a^ Metabolic pathways with significant (*p* < 0.01) and strong (SparCC > 0.7 or < −0.7) correlations; ^b^ number of connections/correlations obtained by SparCC analysis; ^c^ modules are formed by a group of nodes densely connected. A higher number of modules means for a higher number of functions that do not co-occur frequently and slightly interact with other functions; ^d^ the longest distance between nodes in the network. The longest the distance the less robust is the network; ^e^ the average number of connections per node in the network, that is, the node connectivity; ^f^ weighted degree is the sum of the weights of all links attached to node; ^g^ the average clustering coefficient indicates how nodes are embedded in the network. A higher value, together with a higher number of all possible combinations (in parenthesis) among every three nodes, is indicative of a tigter network, with nodes deeply interconnected among them. High values indicate a “small-world” effect or the presence of modules with well-connected nodes but isolated form other modules.

**Table 2:** Topological features of the functional networks under different disturbance factors.

Topological Features	Anti-Tick Immunity		*A. phagocytophilum* Infection		Antimicrobial Peptide	
	Bb-anti-OVA	Bb-anti-PIXR	Ap-uninfected	Ap-infected	sP1	P1
Nodes ^a^	392	404	427	429	425	427
Edges ^b^	19,284	44,362	32,654	28,067	93,162	66,514
Modules ^c^	13	3	5	9	3	3
Network diameter ^d^	6	4	4	4	4	4
Average degree (D) ^e^	98.14	124.15	152.95	130.86	125.41	103.75
Weighted degree (WD) ^f^	74.04	86.51	103.47	85.022	82.62	67.91
Clustering coefficient ^g^	0.783 (777,010)	0.742 (1,087,589)	0.768 (1,603,192)	0.757 (1,302,711)	0.730 (986,125)	0.663 (689,790)

^a^ Metabolic pathway with a significant (*p* < 0.01) and strong (SparCC > 0.7 or < −0.7) correlation; ^b^ number of connections/correlations obtained by SparCC analysis; ^c^ modules are formed by a group of nodes densely connected. A higher number of modules means for a higher number of functions that do not co-occur frequently and slightly interact with other functions; ^d^ the longest distance between nodes in the network. The longest the distance the less robust is the network; ^e^ the average number of connections per node in the network, that is, the node connectivity; ^f^ weighted degree is the sum of the weights of all links attached to node; ^g^ the average clustering coefficient indicates how nodes are embedded in the network. A higher value, together with a higher number of all possible combinations (in parenthesis) among every three nodes, is indicative of a tighter network, with nodes deeply interconnected among them. High values indicate a “small-world” effect or the presence of modules with well-connected nodes but isolated form other modules.

## Supplementary Materials

The following are available online at https://www.mdpi.com/2076-0817/9/4/309/s1, Figure S1: Taxonomic composition of the microbiota of *I. scapularis* ticks under different disturbance factors. Figure S2: Differential microbial diversity of tick microbiota due to disturbance factors. Figure S3: Identification of differential taxa (genera) across all the samples. Figure S4: Differential abundant taxa between sample groups (control vs disturbed) from the experiments. Figure S5: Pathway core across modules of bacterial genera identified by networks of taxa co-occurrence. Figure S6: Topological features of the networks of co-occurring microbial taxa in undisturbed and disturbed tick microbiota through anti-tick immunity. Figure S7: Topological features of the networks of co-occurring microbial taxa in undisturbed and disturbed tick microbiota through *A. phagocytophilum* infection. Figure S8: Topological features of the networks of co-occurring microbial taxa in undisturbed and disturbed tick microbiota through antimicrobial peptide. Figure S9: Results of the attacks on the taxonomic networks. Figure S10: Alpha and beta diversity of the functional profile (pathways) of the microbiome of *I. scapularis* ticks under different disturbance factors. Figure S11: Differential functional profile from all tick microbiome under different disturbance factors. Figure S12: Differential WD of the metabolic pathways among sample groups from the experiments. Figure S13: Polysaccharide biosynthesis pathways affected in response to anti-tick immunity. Table S1: Bacterial taxa (identified until genera level) conforming the taxonomic core across all the samples under study. Table S2: Metabolic pathways included in the functional core across all the samples under study. Table S3: Distribution of the metabolic pathways according to the five clusters identified in the Gneiss analysis (Figure S11).
