# Supplementary material for: Resistance of Tick Gut Microbiome to Anti-Tick Vaccines, Pathogen Infection and Antimicrobial Peptides
Source: Pathogens. 2020 Apr 22;9(4):309. doi: 10.3390/pathogens9040309 (PMC7238099; doi:10.3390/pathogens9040309)
Supplement: Supplementary file 1 [file pathogens-09-00309-s001.zip › Supplementary materials R1.pdf]

Resistance of tick gut microbiome to anti-tick vaccines, pathogen infection and antimicrobial peptides

Agustín Estrada-Peña [1,2,*], Alejandro Cabezas-Cruz [3], Dasiel Obregón [4,5,*]

[1]Faculty of Veterinary Medicine, University of Zaragoza, Spain

[2]Group of Research on Emerging Zoonoses, Instituto Agroalimentario de Aragón (IA2), Zaragoza, Spain

[3]UMR BIPAR, INRAE, ANSES, École Nationale Vétérinaire d'Alfort, Université Paris-Est, Maisons-Alfort, France

[4]Center for Nuclear Energy in Agriculture, University of Sao Paulo, Piracicaba, Brazil

[5]School of Environmental Sciences, University of Guelph, ON, Canada

All authors contributed equally

*Correspondence: Agustín Estarada-Peña: aestrada@unizar.es; Alejandro Cabezas-Cruz: cabezasalejandrocruz@gmail.com; Dasiel Obregón: dasielogv@gmail.com

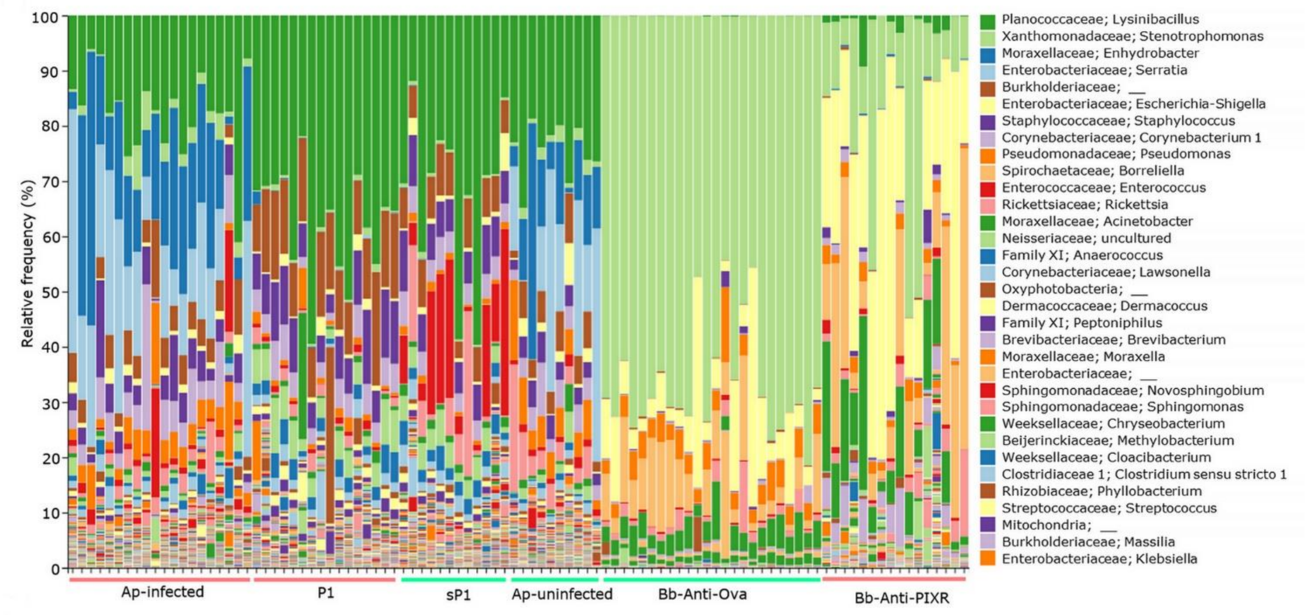

Supplementary Fig. S1. Taxonomic composition of the microbiota of *I. scapularis* ticks under different disturbance factors. **a)** Taxa barplot showing relative abundance of bacterial genera within the tick microbiota. Samples are grouped per experimental groups. Only the topmost abundant taxon (identified at family and genera level) are shown.

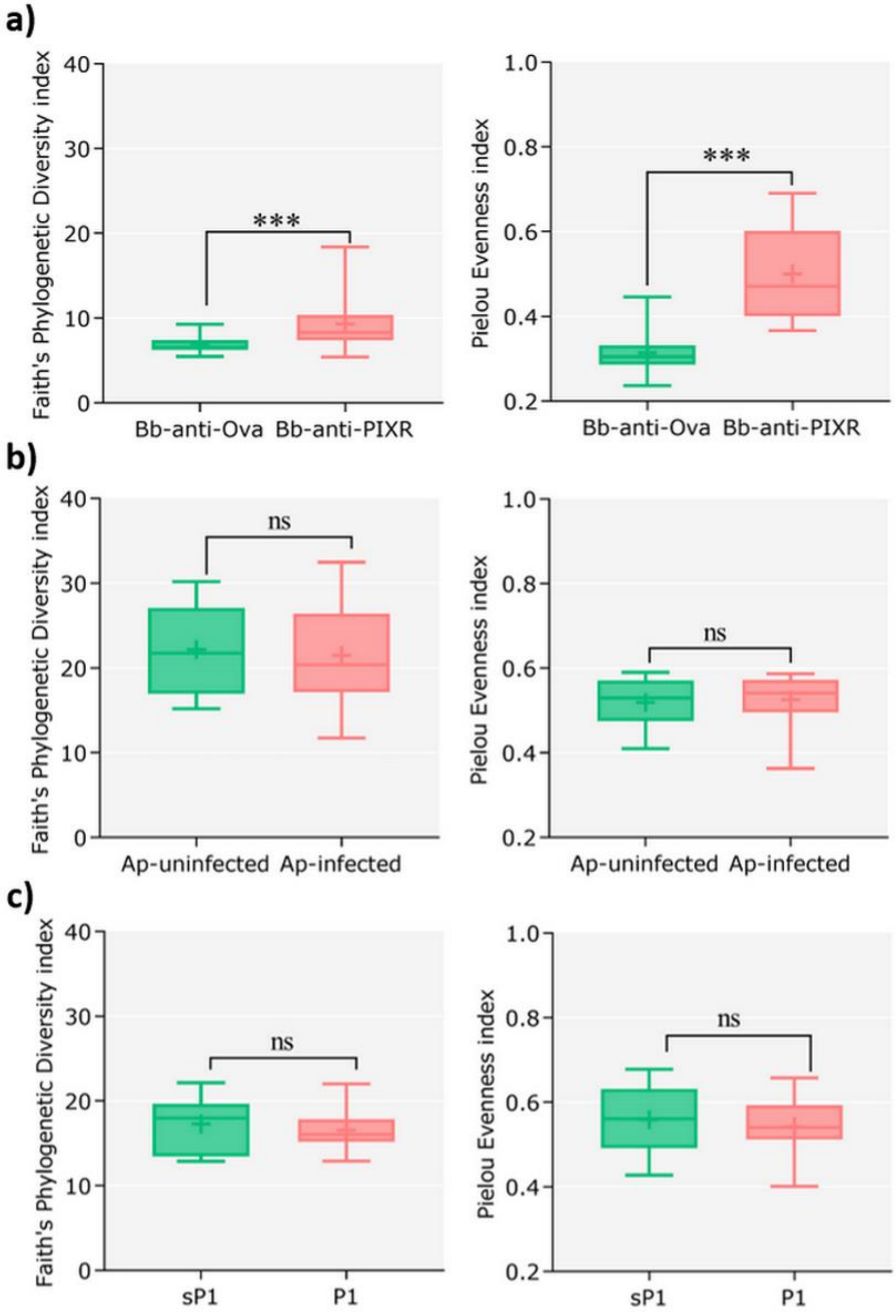

Supplementary Fig. S2. Differential microbial diversity of tick microbiota due to disturbance factors. Comparison of alpha diversity are presented between sample groups from the experiments: **a)** Anti-tick immunity, **b)** *A. phagocytophilum* infection and **c)** Antimicrobial peptide. Richness and evenness were measured by 'Faith PD' and 'Pielou evenness' indexes, respectively, compared by Kruskal-Wallis test (p <0.05*, p <0.01**, p <0.001***).

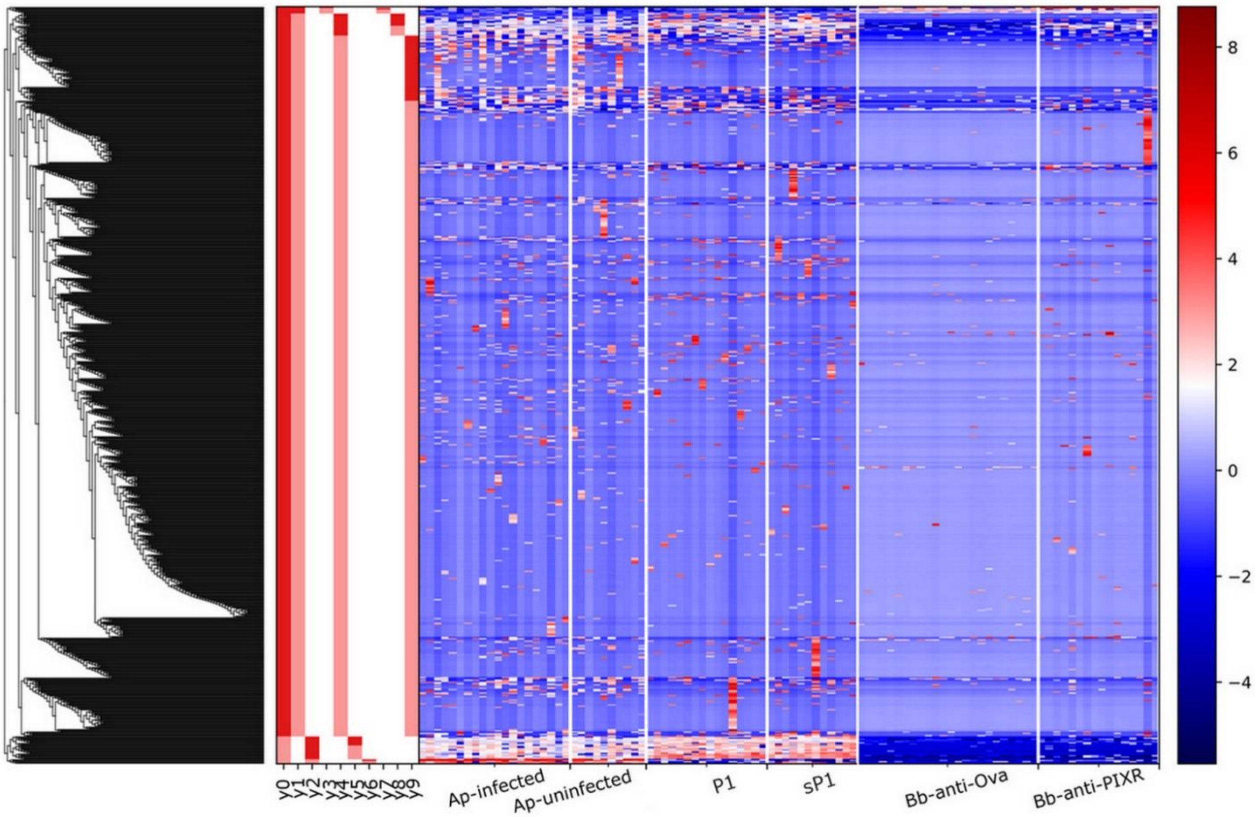

Supplementary Fig. S3. Identification of differential taxa (genera) across all the samples. Dendrogram heatmap resulting from Gneiss analysis. The taxa were clustered based on abundance (log ratio). Nine balances (y0, y1…y9) are calculated based on hierarchical clusters on taxa abundance. Differential taxonomic composition of microbiota of tick larvae (infected with *B. burgdorferi*) and nymphs (infected with *A. phagocytophilum*) are visible according to the main balance (y0).

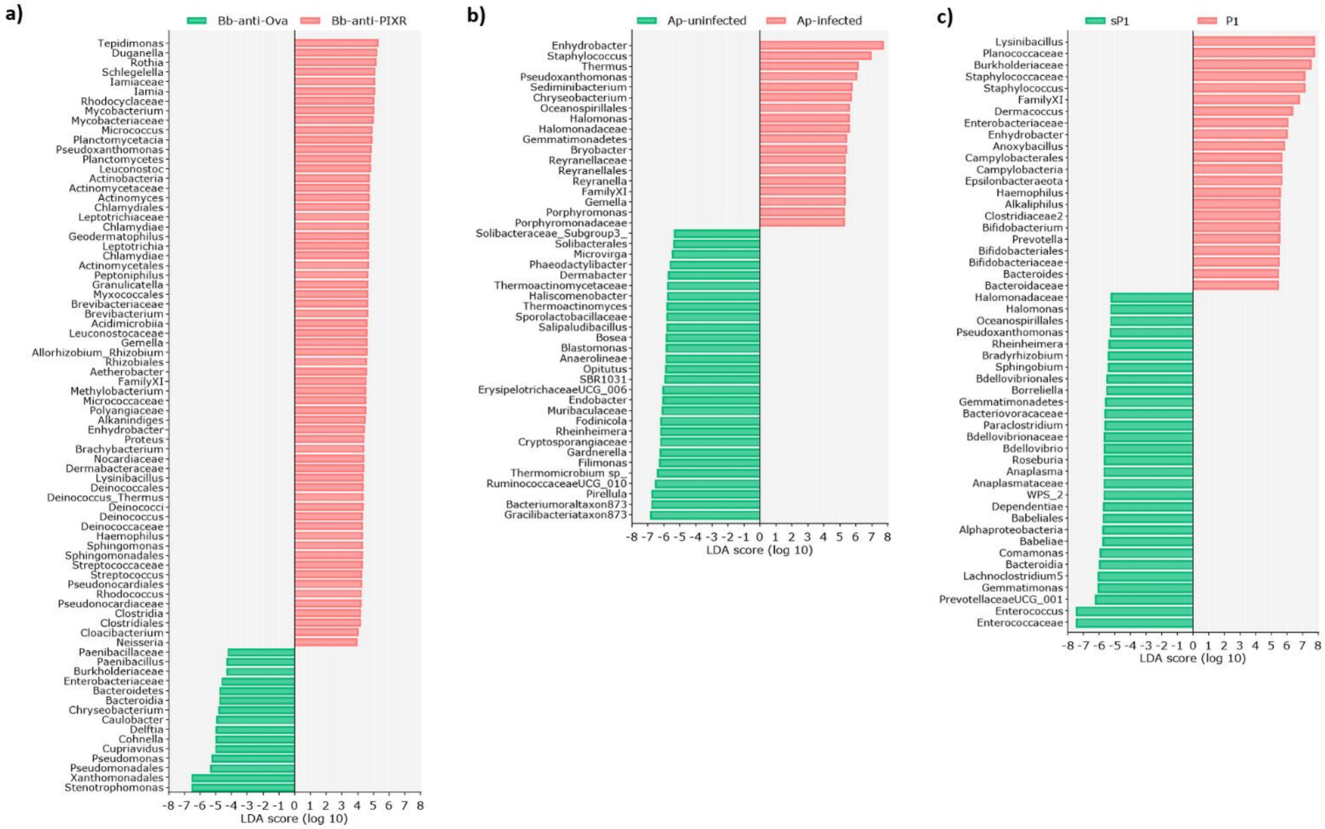

Supplementary Fig. S4. Differential abundant taxa between sample groups (control vs disturbed) from the experiments: **a)** Anti-tick immunity, **b)** *A. phagocytophilum* infection and **c)** Antimicrobial peptide. Differential abundant taxa were identified and ranked by linear discriminant analysis (LDA) effect size (LEfSe).

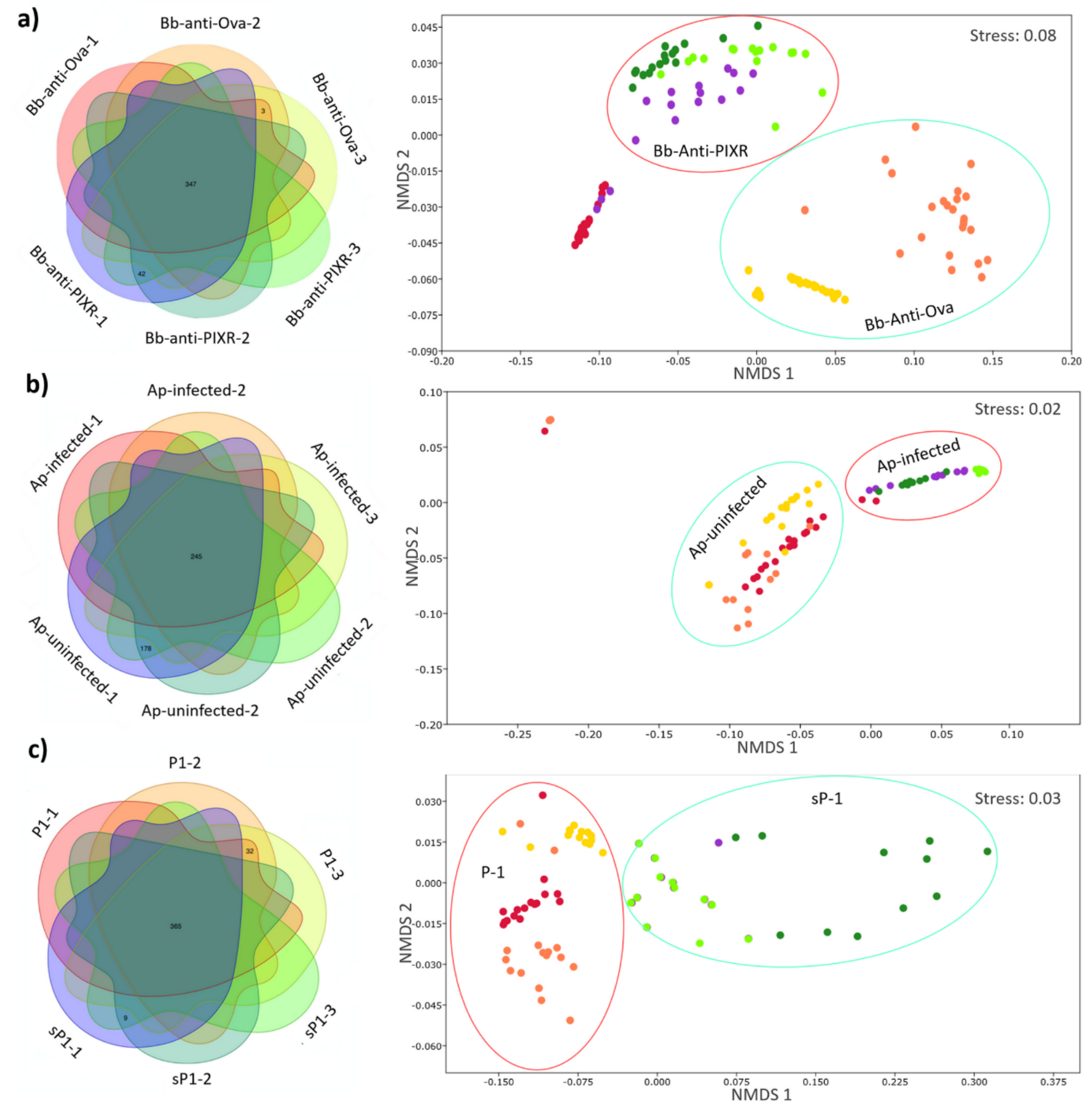

Supplementary Fig. S5. Pathway core across modules of bacterial genera identified by networks of taxa co-occurrence. Venn diagrams show the sharing of the metabolic pathways among 3 randomly selected modules of taxa in each sample group from the experiments: **a)** Anti-tick immunity, **b)** *A. phagocytophilum* infection and **c)** Antimicrobial peptide. Each segment of the Venn diagrams, labeled as ‘1’, ‘2’ or ‘3', represent a randomly selected module of co-occurring taxa that were identified in the co-occurrence networks and then its predicted functional profile was obtained. The six modules represent ~90% of total of identified taxa. Each Venn diagrams is accompanied with a NMDS plot showing shifts in the pathway profile (abundance) over control and disturbed microbiome. Each module profile on the plot is colored with the corresponding color in the Ven diagram, arbitrary ellipsoids are used to facilitate the identification of the sample groups. Spatial ordination based on Bray–Curtis dissimilarity metric.

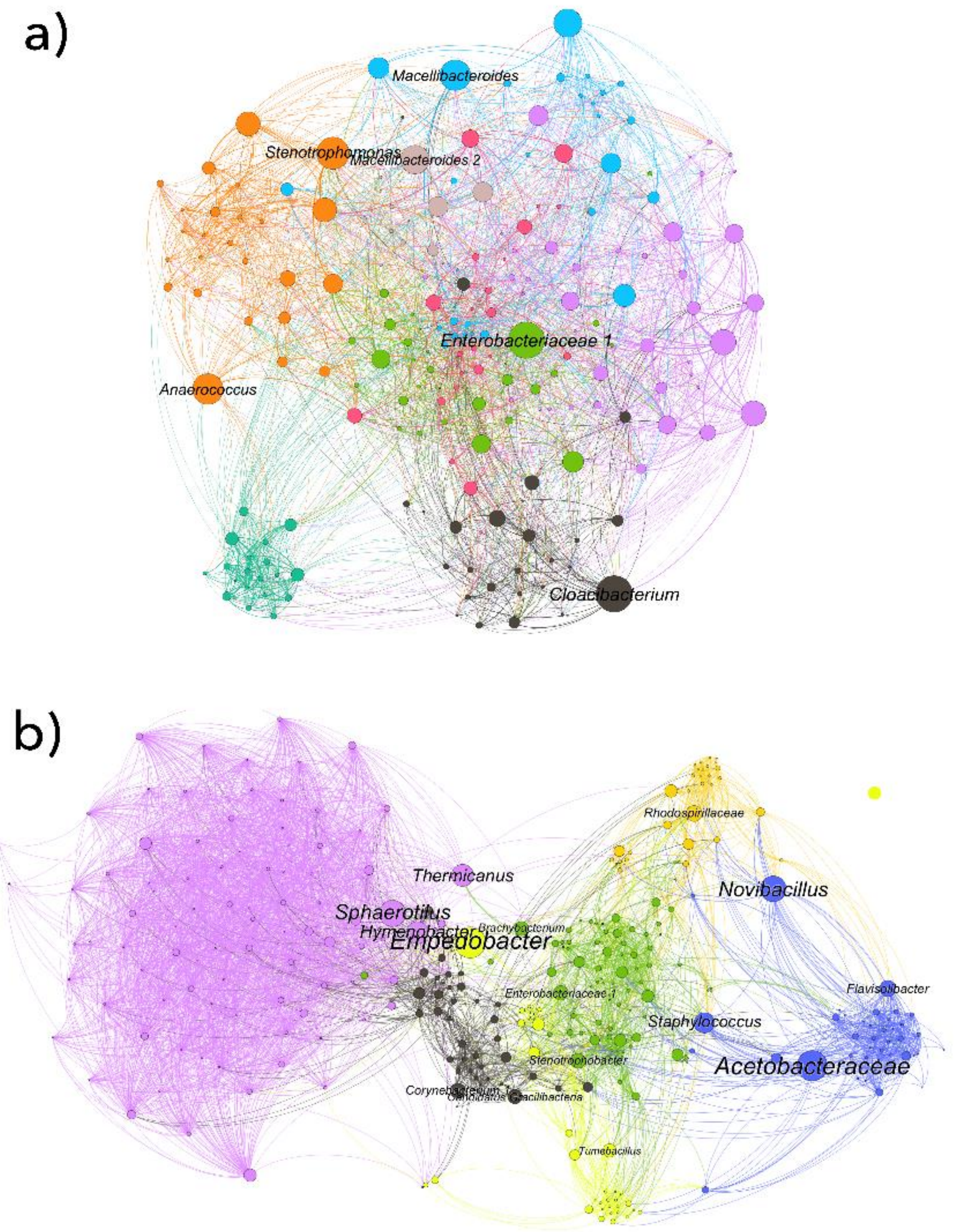

Supplementary Fig. S6. Topological features of the networks of co-occurring microbial taxa in undisturbed and disturbed tick microbiota through anti-tick immunity. Networks are presented by experimental groups: **a)** Bb-anti-Ova, **b)**. Circles (nodes) are bacterial genera and edges the co-occurrence (SparCC >0.7 or <-0.7) between taxa. Colours are random, but circles with the same colour mean for clusters of taxa that co-occur more frequently among them than with other taxa. The size of the circles and the labels is proportional to the betweenness centrality of each taxon in the resulting network.

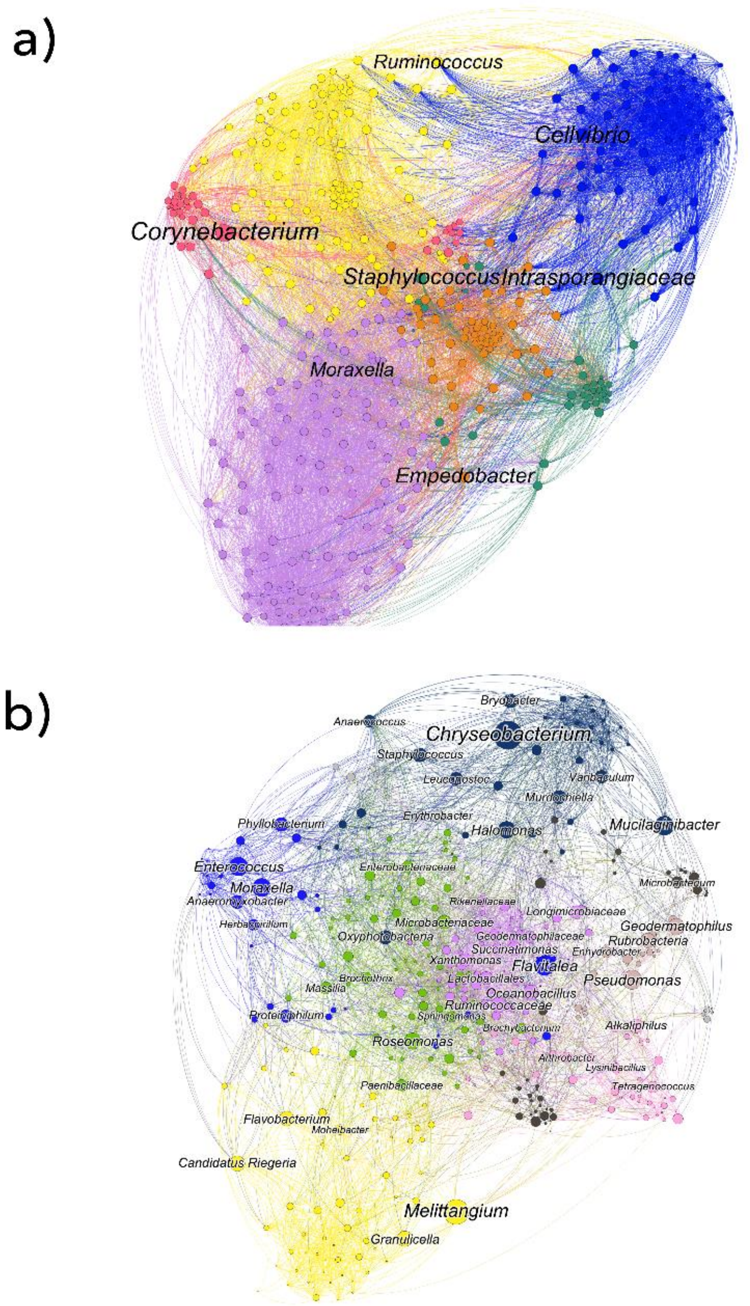

Supplementary Fig. S7. Topological features of the networks of co-occurring microbial taxa in undisturbed and disturbed tick microbiota through *A. phagocytophilum* infection. Networks are presented by experimental groups: **a)** Ap-uninfected, **b)** Ap-infected. Circles (nodes) are bacterial genera and edges the co-occurrence (SparCC >0.7 or <-0.7) between taxa. Colours are random, but circles with the same colour mean for clusters of taxa that co-occur more frequently among them than with other taxa. The size of the circles and the labels is proportional to the betweenness centrality of each taxon in the resulting network.

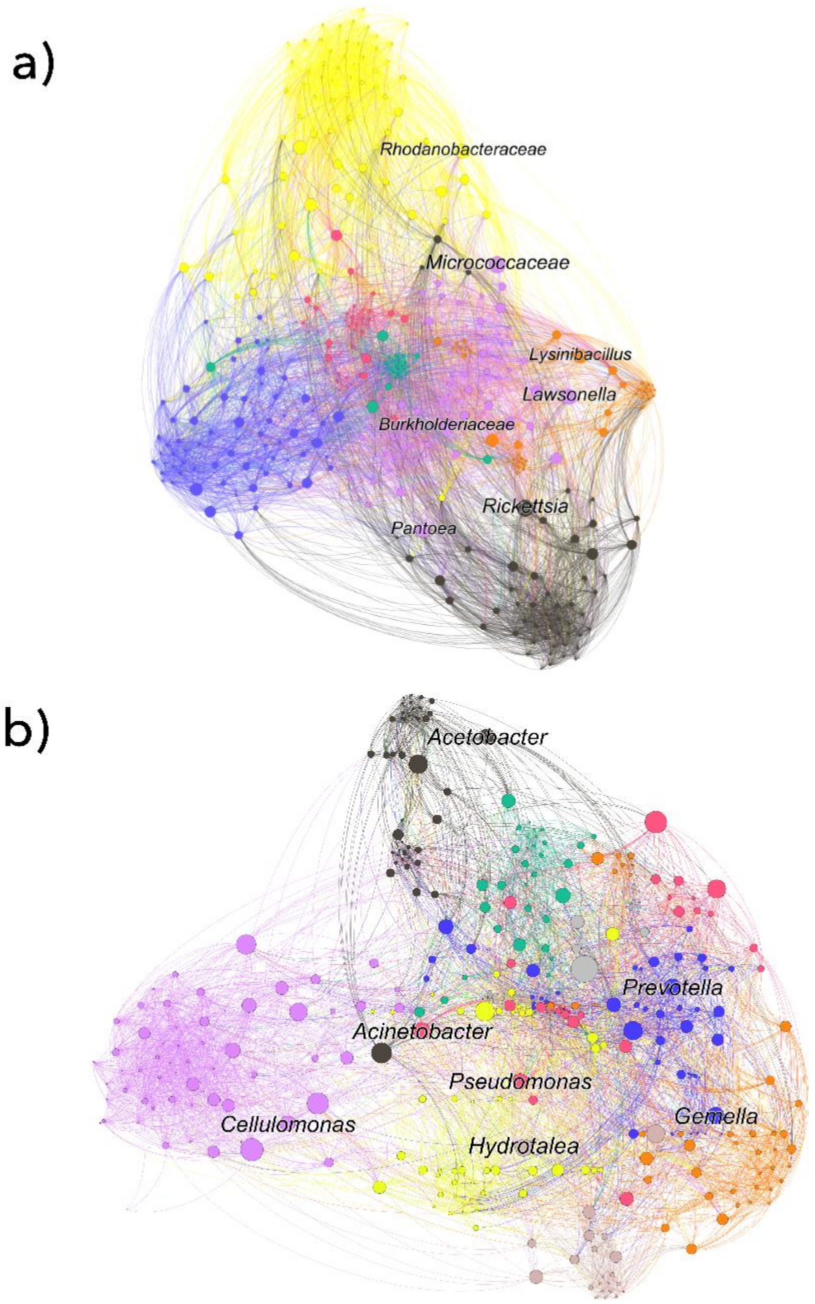

Supplementary Fig. S8. Topological features of the networks of co-occurring microbial taxa in undisturbed and disturbed tick microbiota through antimicrobial peptide. Networks are presented by experimental groups: **a)** sP1, **b)** P1. Circles (nodes) are bacterial genera and edges the co-occurrence (SparCC >0.7 or <-0.7) between taxa. Colours are random, but circles with the same colour mean for clusters of taxa that co-occur more frequently among them than with other taxa. The size of the circles and the labels is proportional to the betweenness centrality of each taxon in the resulting network.

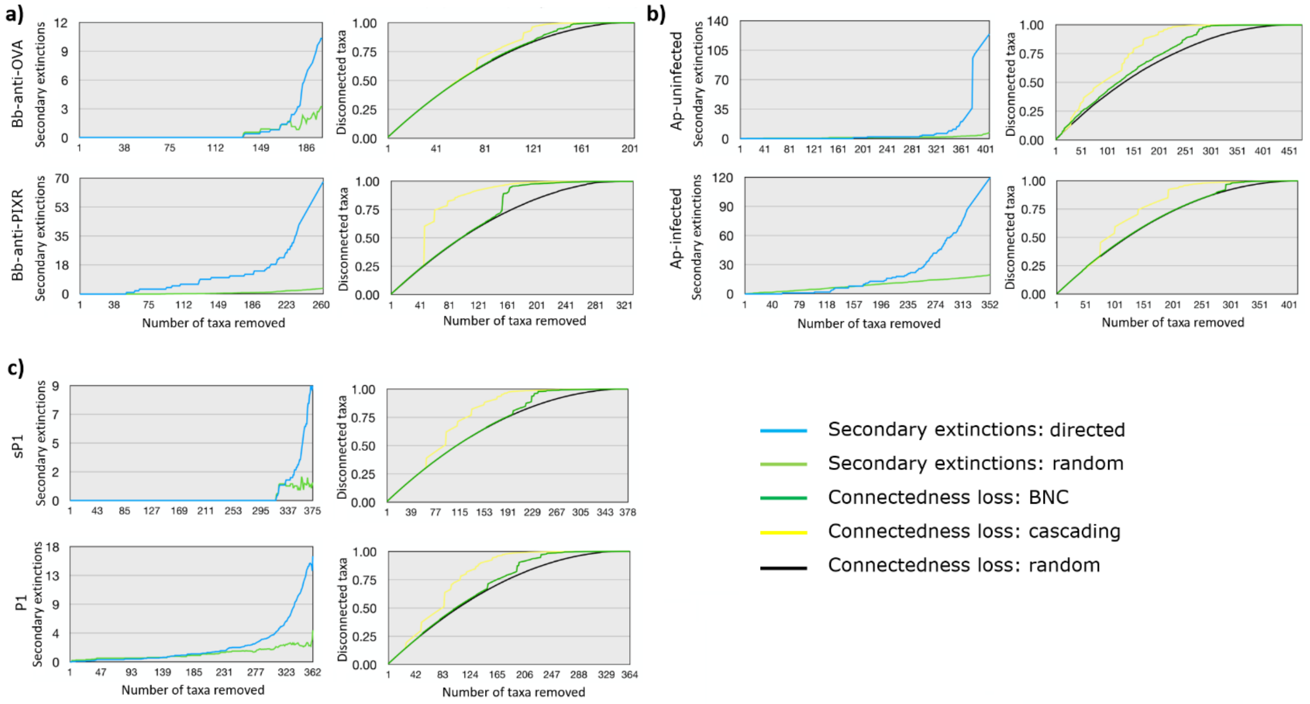

Supplementary Fig. S9. Results of the attacks on the taxonomic networks. Each pair of charts are the results obtained for the calculation of secondary extinctions after individual node removal, randomly or following ascending order of centrality (left) and the features of disconnections of taxa in the network (right). The legend at the right column includes the loos of connectivity when taxa are removed according to the increasing value of centrality (loss_connect_BNC), the loss of connectivity as a cascading effect (loss_connec_Cascading) and the loss of connectivity if taxa are randomly removed (loss_connec_Random). **a)** Bb-anti-Ova, Bb-anti-PIXR; **b)** *A. phagocytophilum* uninfected; *A. phagocytophilum* infected; **c)** Antimicrobial peptide sP1, Antimicrobial peptide P1.

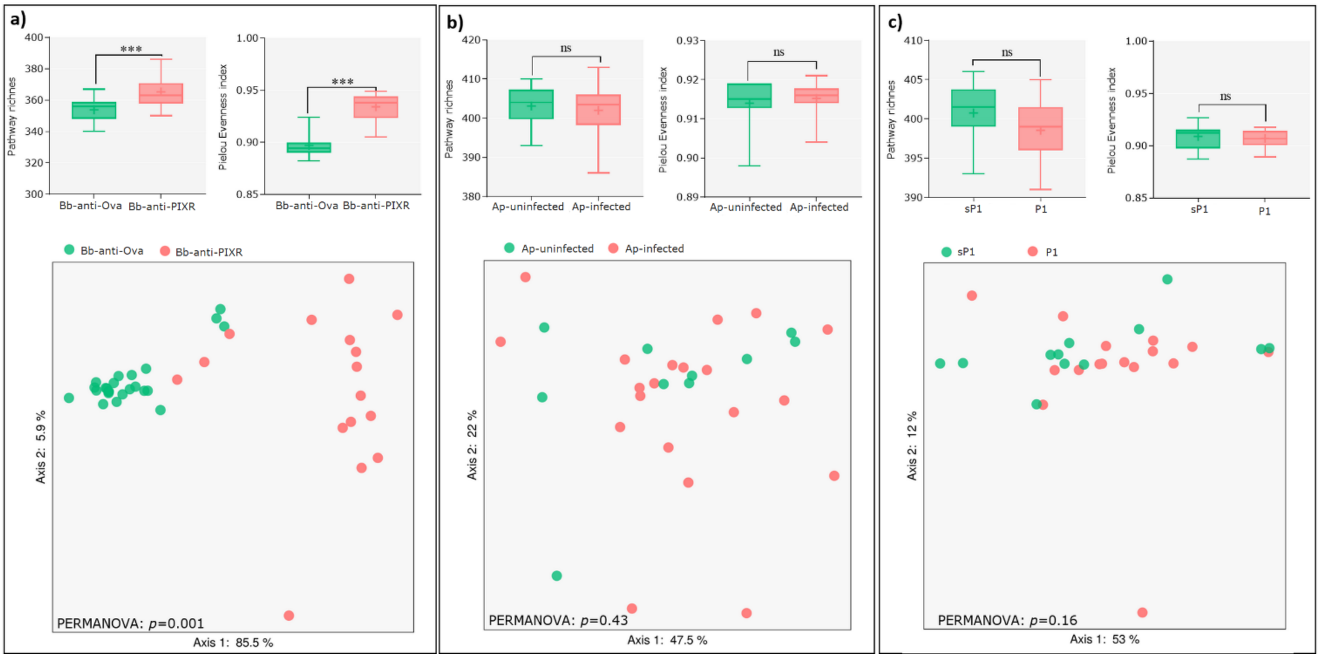

Supplementary Fig. S10. Alpha and beta diversity of the functional profile (pathways) of the microbiome of *I. scapularis* ticks under different disturbance factors. Comparisons of alpha and beta diversity are presented between sample groups from the experiments: **a)** Anti-tick immunity, **b)** *A. phagocytophilum* infection, and **c)** Antimicrobial peptide. Richness and evenness (alpha diversity) were measured by 'Faith PD' and 'Pielou evenness' indexes, respectively, and compared by Kruskal-Wallis test (p <0.05*, p <0.01**, p <0.001***). The beta diversity (PCoA plots) was measured by 'Weighted UniFrac metric' and compared between each pair of sample groups by Permutational multivariate analysis of variance (PERMANOVA).

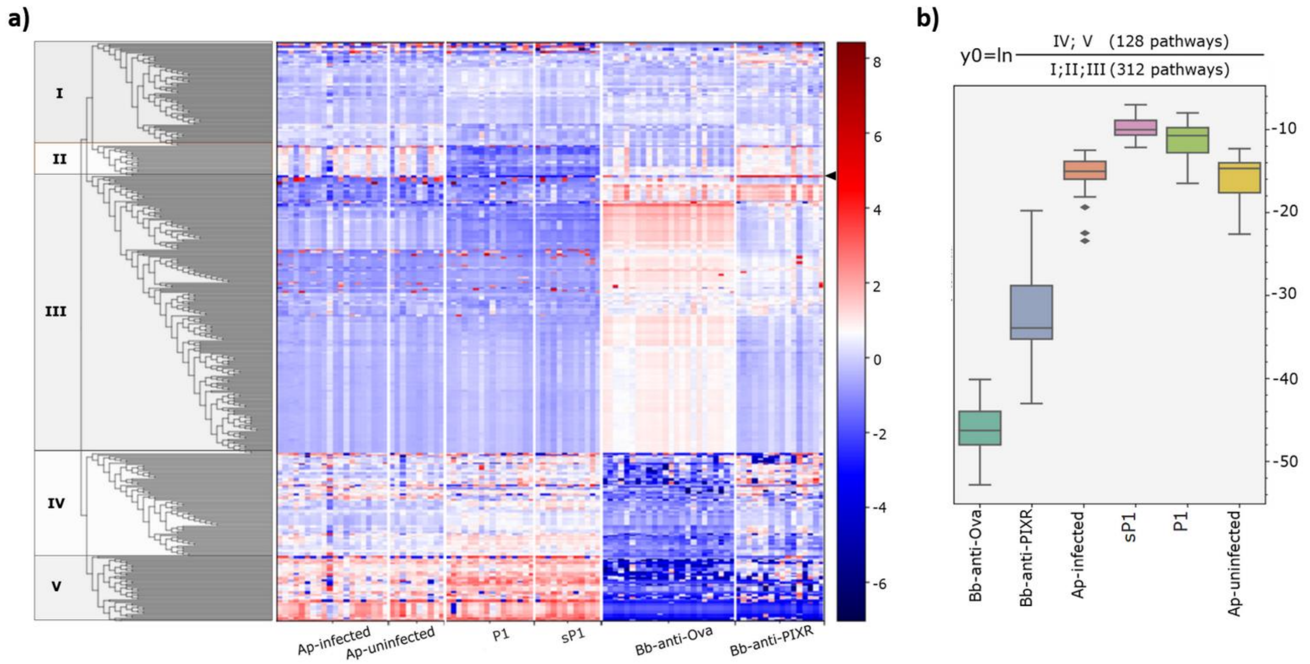

Supplementary Fig. S11. Differential functional profile from all tick microbiome under different disturbance factors. **a)** Dendrogram heatmap showing the log abundance of pathways in each sample groups according to Gneiss analysis. The bifurcating tree was generated from hierarchical clustering of pathways based on abundance (log). Nine balances (not showed) on the relation of that clusters were generated, differences in relative abundance between sampled groups are visible according to the clutters, **e)** The main balance (y0) evidenced the differential pathway abundance profiles from microbiomes of *Ixodes scapularis* tick larvae (infected with *B. burgdorferi*) and nymphs (infected with *A. phagocytophilum*), respectively.

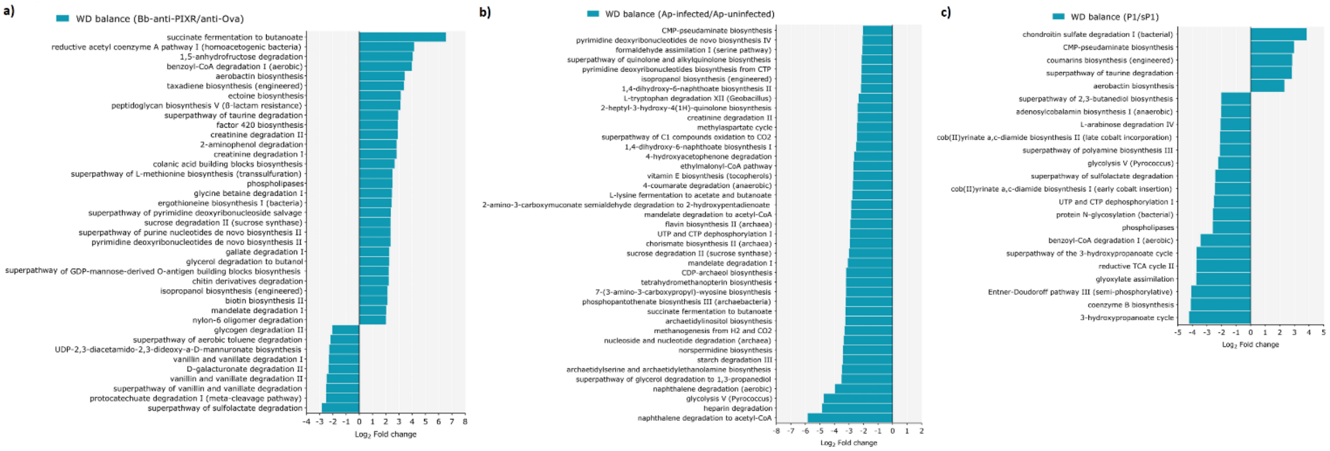

Supplementary Fig. S12. Differential WD of the metabolic pathways among sample groups from the experiments: **a)** Host immunity, **b)** *A. phagocytophilum* infection, and **c)** Antimicrobial peptide. Histograms show the 2fold change of WD observed between control and disturbed tick microbiomes, as resulted from the network of co-occurring pathways. Only pathways that increase (or decreased) in at least twice the value of WD (2-fold-change: log2 = 2) are shown.

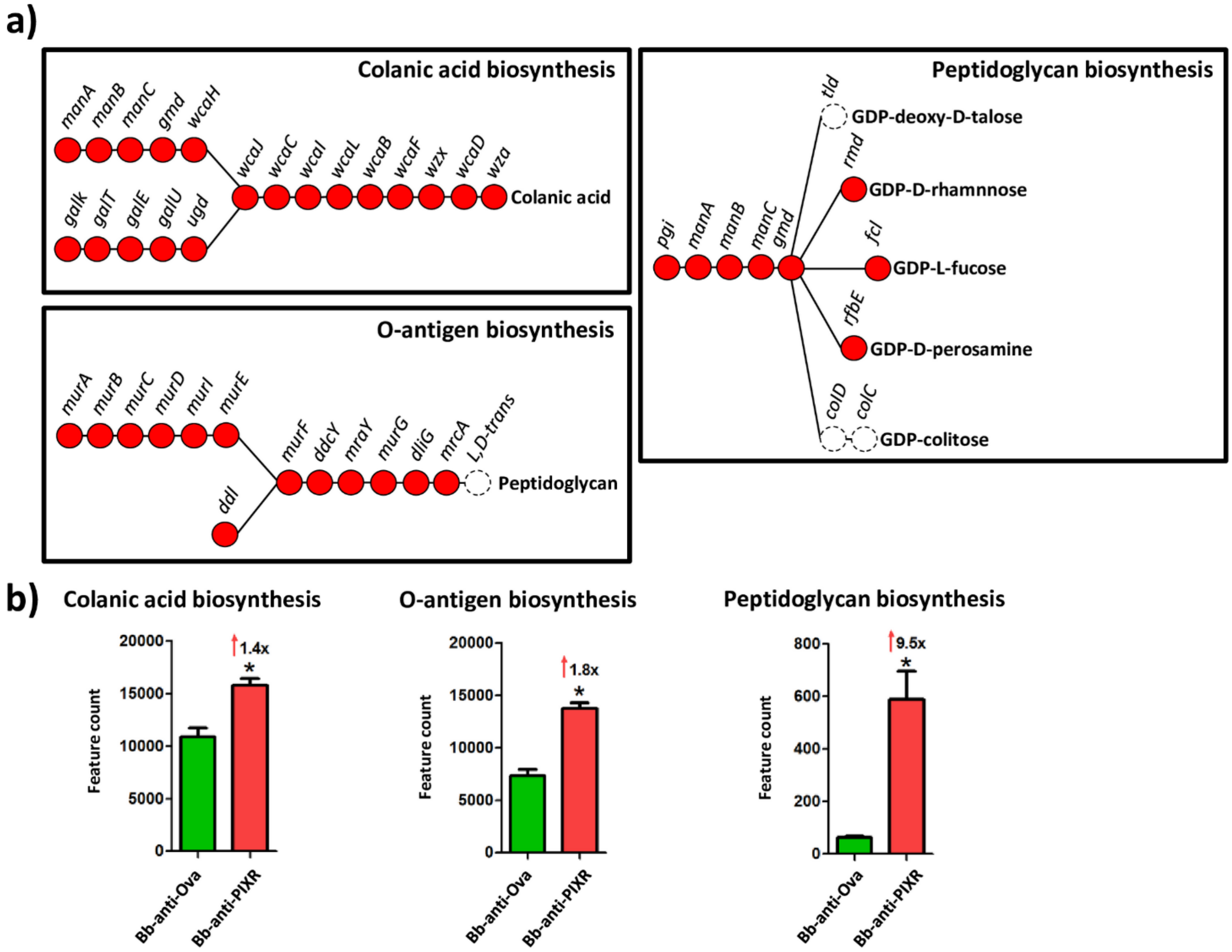

Supplementary Fig. S13. Polysaccharide biosynthesis pathways affected in response to anti-tick immunity. **a)** polysaccharide biosynthesis pathways for which changes in WD were higher than 2-fold-change (log2 = 2) in response to anti-tick immunity. Pathway reconstruction was performed using KEGG reference pathways and KEGG enzymes predicted by PICRUSt. **b)** The 'feature count' of each pathway in the datasets 'Bb-anti-Ova' (n=24) and 'Bb-anti-PIXR' (n=16) is presented. Feature count values were compared by Mann Whitney test (p <0.05*).
